# Requirements for a Robust Animal Model to Investigate the Disease Mechanism of Autoimmune Complications Associated With ARF/RHD

**DOI:** 10.3389/fcvm.2021.675339

**Published:** 2021-05-05

**Authors:** Rukshan A. M. Rafeek, Suchandan Sikder, Adam S. Hamlin, Nicholas M. Andronicos, David J. McMillan, Kadaba S. Sriprakash, Natkunam Ketheesan

**Affiliations:** ^1^School of Science and Technology, University of New England, Armidale, NSW, Australia; ^2^Department of Medicine and Surgery, Chattogram Veterinary and Animal Sciences University, Chattogram, Bangladesh; ^3^School of Science, Technology, Engineering and Genecology Research Centre, University of the Sunshine Coast, Maroochydore, QLD, Australia; ^4^Queensland Institute of Medical Research Berghofer, Brisbane, QLD, Australia

**Keywords:** animal model, acute rheumatic fever, rheumatic heart disease, sydenham chorea, lewis rats, autoimmunity, Group A streptococcus

## Abstract

The pathogenesis of Acute Rheumatic Fever/Rheumatic Heart Disease (ARF/RHD) and associated neurobehavioral complications including Sydenham's chorea (SC) is complex. Disease complications triggered by Group A streptococcal (GAS) infection are confined to human and determining the early events leading to pathology requires a robust animal model that reflects the hallmark features of the disease. However, modeling these conditions in a laboratory animal, of a uniquely human disease is challenging. Animal models including cattle, sheep, pig, dog, cat, guinea pigs rats and mice have been used extensively to dissect molecular mechanisms of the autoimmune inflammatory responses in ARF/RHD. Despite the characteristic limitations of some animal models, several rodent models have significantly contributed to better understanding of the fundamental mechanisms underpinning features of ARF/RHD. In the Lewis rat autoimmune valvulitis model the development of myocarditis and valvulitis with the infiltration of mononuclear cells along with generation of antibodies that cross-react with cardiac tissue proteins following exposure to GAS antigens were found to be similar to ARF/RHD. We have recently shown that Lewis rats injected with recombinant GAS antigens simultaneously developed cardiac and neurobehavioral changes. Since ARF/RHD is multifactorial in origin, an animal model which exhibit the characteristics of several of the cardinal diagnostic criteria observed in ARF/RHD, would be advantageous to determine the early immune responses to facilitate biomarker discovery as well as provide a suitable model to evaluate treatment options, safety and efficacy of vaccine candidates. This review focuses on some of the common small animals and their advantages and limitations.

## Introduction

The concept of comparative medicine developed based on the theory that animal species share physiological, anatomical and behavioral characteristics similar to human (1). This concept led to the use of different model organisms in all fields of biomedical research (2) and they continue to play a vital role in translational research for the advancement of human and animal health. The use of animal models to investigate human disease has its origins over 2,400 years ago. By the beginning of the twentieth century the use of animal models became more experimental rather than observational (1). Animals have contributed immensely in elucidating the disease mechanisms and the development of therapeutics including vaccines. An animal model, in which the immunopathological mechanisms or outcome of disease resembles those that occur in humans, is a logical adjunct to investigate human diseases. Thus, in this review we summarize the current animal models available to investigate the pathogenesis of Acute Rheumatic Fever (ARF), Rheumatic Heart Disease (RHD) and associated post-streptococcal autoimmune complications.

Post-streptococcal autoimmune disorders are complex immune mediated disease mostly affecting children and young adults following exposure to Group A streptococcal (GAS) infection. These includes ARF, RHD, Sydenham Chorea (SC) and possibly, pediatric autoimmune neuropsychiatric disorders associated with streptococcal infections (PANDAS) (3–5). After 1–3 weeks of an untreated GAS infection, ~1–3% of individuals develop non-suppurative post streptococcal complications including ARF, which may lead to RHD and cardiac failure (6). ARF affects multiple organs and primarily involve the joints, skin, brain and the heart. Except for cardiac damage most other manifestations are transient. After the initial or repeated episodes of ARF, about 30–45% of patients develop RHD (5) which poses an important public health problems in low to middle-income countries, and First Nation Peoples of high-income countries. Indigenous Australians (Aboriginal and Torres Strait Islander people) and New Zealanders (Māori and Pacific Islander populations) have among the highest rates of ARF in the developed countries (5, 7). RHD is the most common acquired cause of cardiac damage (8) affecting children between the ages of 5 and 15 years old (9). A gender propensity for ARF has not been widely observed although some studies have found RHD to be prevalent among females (10). The epidemiology of ARF/RHD is highly diverse and is relatively rare where access to modern medical care is readily available. However, it has not been completely eradicated with annual incidence of ARF varying from <0.5/100,000 in developed countries to >100/100,000 in developing countries (11). It is estimated that annually, approximately half a million new ARF cases are diagnosed globally (11). On the other hand, the overall prevalence of RHD is highest in sub-Saharan Africa, South Asia and Oceania. In 2015, 33.4 million people were reported to be have RHD with ~297,300–337,300 deaths in RHD endemic regions. In non-endemic regions it was 221,600 cases (12).

Variety of host, bacterial, socioeconomic and environmental factors contribute to the prevalence and incidence of ARF/RHD (5). Environmental factors includes climatic factors, sanitation, poor hygiene, overcrowding and house hold conditions (5). In addition better living conditions led to decrease in the incidence of ARF/RHD (13, 14). Malnutrition and poverty are two other important factor among children contributing to repeated exposure to streptococcal and the spread of infection (13, 15). Poor healthcare system due to low socioeconomic status and inadequate awareness of the disease in the community leads to misdiagnosis or late diagnosis and treatment of GAS infection and ARF/RHD (5, 14, 16). In addition, a strong predisposition of genetic factors including genetic polymorphisms in many human leukocyte antigen (HLA) class II alleles in the development of ARF/RHD have also been described (17).

## Pathogenesis of ARF/RHD

The pathophysiology of post-streptococcal complication is not fully understood, however antigenic mimicry between GAS antigens and host proteins is partly considered as factor that triggers autoimmunity. It may also be affected by several environmental, genetic and socioeconomic factors. Although an autoimmune process has long been considered to be responsible for the initiation of ARF/RHD, it is only in the last few decades that the mechanisms involved in the pathogenesis of this post streptococcal conditions have been unraveled partly due to experimentation on animal models. Studies have shown that molecular mimicry of streptococcal antigens enable the generation of antibodies that bind to both GAS antigens and cross-react with host tissue proteins including cardiac myosin, collagen I and IV, tropomycin, laminin, vimentin, and keratin (18).

Further studies have demonstrated that human collagen IV, one of the major components of the basal membrane, a layer of extracellular matrix secreted by epithelial cells, can also be involved in the pathogenesis of ARF/RHD by acting as an autoantigen after forming a complex with GAS antigens (19). Several studies have demonstrated that GAS strains are capable of binding and aggregating to human collagen (6, 20–22). Collagen IV binds to cells and other molecules via an N- terminal Cyanogen Bromide fragment 3 (CB3) (19). GAS binds to CB3 of collagen via the octapeptide (AXYLZZLN) epitope of M protein and aggregate to form an antigenic complex with human collagen IV (19). The octapeptide region of M protein, which interact with collagen, is designated as PARF (peptide associated with rheumatic fever). The autoantigenicity of the M protein-collagen complex induces ARF/RHD. Higher levels of anti-collagen antibodies were found in the sera of ARF patients than healthy controls (22). In addition studies showed that injection of mice with GAS proteins also induce a collagen autoantibody response. However, these antibodies did not cross-react with the respective M protein. This observation leads to the understanding that the collagen autoimmunity caused by PARF motif of M protein does not depend on molecular mimicry (22).

## Animal Models of ARF/RHD

Post streptococcal autoimmune complications including ARF/RHD is uniquely a human condition and humans are the only host and reservoir for GAS. Thus, modeling post-streptococcal autoimmune complications in animal is challenging. However, animals are the only experimental models used to investigate the characteristic signs, pathogenesis and pathophysiology specific to ARF/RHD. Animals including guinea pigs, rabbits, pigs, sheep, goats, cattle, cats, dogs, and non-human primates have been used as experimental model to understand the disease mechanism of ARF/RHD and to investigate the rheumatogenic potential of GAS M proteins (23–36). In the last two decades these animals were replaced by mice and rats due to lower costs, ease of handling and observation of pathological, immunological and functional changes comparable to ARF/RHD patients (Table 1).

**Table 1:** Immunopathological changes in small animals and rodents investigated as experimental model for post streptococcal complications.

	**Antigens (route of injection)**	**Histological changes**	**Antibody response**	**T-cell and cytokine response**	**Tissue cross reactivity**	**References**
**Cardiac pathology**						
**Mice**	Cell wall fragments of GAS (*i.p*.)	**Myocarditis, valvulitis** MNCs, anitschkow cell, PMNCs	Anti-GAS IgG collagen IV reactive IgG	N/A	Basement membrane collagen	(20, 43)
	Recombinant proteins/peptides of GAS (*f.p., s.c*.)	**Myocarditis, valvulitis**	Anti-GAS IgG	CD4+ (M)	Myosin	(77)
**Lewis rats**	Whole GAS and/or SDSE (*f.p., s.c*.)	**Myocarditis, valvulitis** monocyte, fibroblast, aschoff like cell lymphocyte, macrophages	Anti-streptococcal IgG Anti-myocardial IgG, antistreptolysin O	CD3+ (M), CD68+ (M), IFN-γ 🠙 (B), IL-17A 🠙 (B), IL-4 🠙 (B)	Myocardial protein, valvular protein, cardiac myosin, collagen	(31, 32, 35, 36)
	Recombinant proteins/peptides of GAS and/or SDSE *(f.p., s.c.)*	**Myocarditis, valvulitis** T-cell, MNCs, PMNCs, anitschkow cell	Anti-myosin IgG Anti- collagen IgG	CD3+ (M), CD4+ (M), CD8+ (M), CD68+ (M), IFN-γ 🠙, IL-17A 🠙, IL-4 🠙	Myosin, valvular protein, collagen	(26, 27, 30, 34–36)
	Serum from GAS exposed rats (*s.c*.)	**Myocarditis, valvulitis** T-cell, MNCs, PMNCs, anitschkow cell	Anti-myosin IgG	CD4+ (M)	Myosin, valvular protein, collagen	(36)
**Guinea pig**	Whole GAS (*s.c., f.p., i.m., i.v*.)	**Myocarditis, valvulitis** B-cell, macrophages, MNCs, fibroblast, cytotoxic lymphocytes	Anti-streptococcal IgG	N/A	Cardiac myofibre, sarcolemma	(23, 25)
	Cell wall fragments of GAS (*s.c., f.p., i.m*.)	**Myocarditis, valvulitis** B-cell, macrophages, fibroblast, cytotoxic lymphocytes	Anti-streptococcal IgG	N/A	Cardiac myofibre, sarcolemma	(25)
**Rabbit**	Whole GAS (*s.c., f.p., i.m., i.v., i.d., i.p*.)	**Myocarditis, valvulitis** lymphocyte, MNCs, leukocyte, aschoff bodies, fibroblast, fibrin, collagen	N/A	N/A	Skeletal muscle	(23–25)
	Cell wall fragments of GAS (*s.c., f.p., i.m., i.v., i.d*.)	Myofibrosis with degeneration of sarcoplasm, lymphocyte, macrophages, granulocyte	Anti-myosin IgG, Anti-sarcolemmal Ig	T-cell, IL-6 🠙, C3 (CV)	Sarcolemmal membrane protein, myosin	(25, 28, 29, 78)
**Neurobehavioral changes**						
**Mice**	Whole GAS (*s.c., i.n*)	**Antibody deposition** deep cerebellar nuclei globus pallidum, thalamus, periventricular areas	Anti-GAS IgG	CD4+, CD68+Iba1+ Th1 🠙, Th17 🠙, IL-17A 🠙, IFN-γ 🠙(B)	N/A	(57, 61, 62, 67)
	Serum from GAS exposed mice (*s.c*.)	**Antibody deposition** hippocampus periventricular area	Anti-GAS IgG1	IL-4 🠙 (B)	N/A	(58)
**Lewis rats**	Whole GAS (*s.c*.)	**Antibody deposition** striatum, thalamus, and frontal cortex	Anti-GAS IgG Anti-dopamine IgG	N/A	Dopamine D1R and D2L receptors	(59)
	Serum from GAS exposed rats (*s.c*.)	**Antibody deposition** striatum	Anti-GAS IgG	N/A	Dopamine D1R and D2L, serotonin transporter	(60)

*f.p., foot pad; i.v., intra venous; i.m., intra muscular; i.d., intra dermal; s.c., sub cutaneous; i.p., intra peritoneal; CV, Cardiac Valve; C, Complement; B, Blood; GAS, Group A streptococci; IFN, Interferon; IgG, Immunoglobulin G; IL, Interleukin; MNC, Mononuclear Cell; M, Myocardium; SDSE, Streptococcus dysgalactiae subspecies equisimilis; PMNC, Polymorphonuclear cells; Th, helper T-cell; TNF, Tumor necrosis factor; 🠙, Elevated; N/A, Not Assessed*.

The early experiments on rheumatic myocarditis were carried out in rabbits based on the hypothesis that ARF/RHD was caused either by direct streptococcal infection or by direct damage to heart tissues by streptococcal toxins. However, none of the rabbits showed similar pathology to rheumatic myocarditis in these studies (23). A study by Gross et al. as early as in 1929 examined the development of rheumatic myocarditis induced by live and killed streptococci isolated from patients with ARF/RHD in seven different animals including rabbits, guinea pigs, dogs, cats, swine, sheep, and calves. These studies failed to induce myocarditis in any of these animals (23). However, some rabbits showed accumulation of lymphocytes and mononuclear cells in their myocardium, low-grade pericarditis with mononuclear cells, acute focal interstitial myocarditis and large, irregular, thrombotic mass on the posterior cusp of the mitral valve. Similarly, guinea pigs showed focal interstitial accumulations of lymphocytes and large mononuclear cells in the myocardium, whereas dogs and cats had no gross or microscopic pathological cardiac lesions. Only one of the pigs in the study developed transient arthritis which disappeared after only a few days. The only positive pathological finding in sheep was a few interstitial foci of lymphocytes and mononuclear cells in the myocardium of the left ventricle (23).

To investigate the role of cellular immune response in RHD, Yang et al. (25) injected Guinea pigs with heat killed GAS and/or GAS M protein. Animals developed valvulitis and myocarditis with infiltration of T and B cells, macrophages and fibroblast into the myocardium and mitral valve (25). Myocardial and endothelial damage due to infiltration of granulocytes, macrophage and lymphocytes were observed in New Zealand White Rabbits injected with GAS M proteins (28, 29, 37) (Table 1). In addition, GAS pharyngeal spray on non-human primate (rhesus monkey, *Macaca mulatta*) induced typical RHD lesions as well as evidence of myocarditis and valvulitis along with infiltration of lymphocytes, histiocytes, Anitshkow cells, and plasma cells (38). Later, subcutaneous injection of GAS membrane antigens to rhesus monkeys' showed similar histological changes with endocardial and sub endocardial infiltration of mononuclear cells (39). Despite numerous attempts, relevant animal model for ARF/RHD still remains elusive (Table 1).

## Rodent Models of ARF/RHD

Rodent models due to ease of handling, small body size, large litter sigs, short life span and cost are considered ideal for biomedical research (1). The Swiss-Webster mice were the first rodent model of ARF/RHD (32). These mice developed cardiac lesions similar to ARF when infected with GAS cell wall fragments. MRL+*/*+ mice injected with *N*-terminal peptides of GAS M5 protein developed myocarditis (40). Moreover, myocarditis and CD4+ lymphocyte infiltration was detected in BALB/c (41, 42), Swiss mice (43), A/J mouse (44) and DBA/2 (45) mouse strains following the injection of GAS antigens and/or cardiac myosin. A more robust animal model for ARF/RHD was developed by immunizing Lewis rats with GAS M protein (26, 46). Upon injection of GAS antigens, or cardiac myosin, animals developed myocarditis and/or valvulitis similar to patients with ARF/RHD with antibody and T-cell responses that cross-reacted with host cardiac proteins.

## Rat Autoimmune Valvulitis Model of ARF/RHD

Lewis rats were used to scrutinize myocarditis by injection of cardiac myosin. Marked cellular infiltration consisting of mononuclear cells, neutrophils, fibroblasts, and multinucleated giant cells were observed in the experimental allergic myocarditis (EAM) (47). Quinn et al. in 2001 developed the Lewis rat autoimmune valvulitis (RAV) model following exposure to streptococcal antigens to investigate the pathogenesis of ARF/RHD (26). This Lewis rat model has become the dominant animal model used to investigate the pathogenesis of ARF/RHD and to determine the safety of experimental GAS vaccine candidates (27, 30, 33–36).

**Figure 1:**
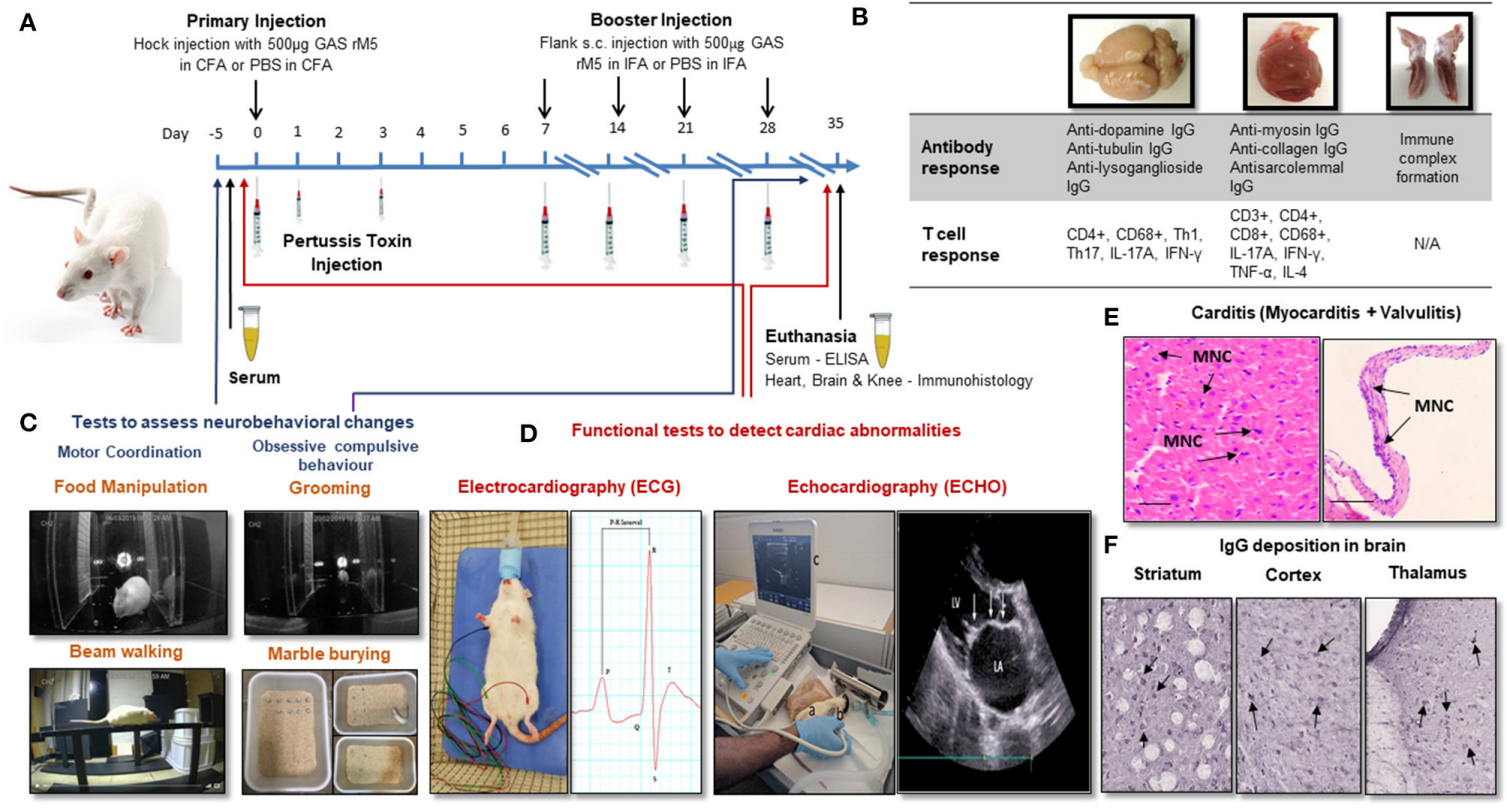
Procedures for the induction of carditis and neurobehavioral changes in Lewis rats. **(A)** Carditis, valvulitis and neurobehavioral changes can be induced by injection of Lewis rats with GAS antigens followed by *Bordetella pertussis* toxin injection and booster injection of GAS antigens. **(B)** Antibody and T cell response can be assessed in brain, heart and knee joints following injection of GAS antigens. **(C)** Standard behavioral tests to assess neurobehavioral changes following exposure to GAS antigens. **(D)** ECG and ECHO changes will demonstrate impairment cardiac function. **(E)** Characteristic mononuclear cell infiltration in the myocardium and valvular tissue (arrows) can be demonstrated in the histological sections of cardiac tissue from rats injected with GAS antigens. **(F)** IgG deposition can be demonstrated in sections of brain following incubation with sera from rats exposed to GAS antigens. CFA, Complete Freund's Adjuvant; IFA, Complete Freund's Adjuvant; PBS, Phosphate Buffered Saline; GAS rM5, Recombinant M5 protein of Group A streptococcus; ELISA, Enzyme Linked Immunosorbent Assay; Th, Helper T-cell; TNF, Tumor Necrosis Factor; IFN, Interferon; IgG, immunoglobulin G; IL, interleukin; MNC, Mononuclear Cell.

Lewis rats immunized with recombinant M6 (rM6) protein demonstrated valvulitis and focal myocarditis, which were histologically similar to pathological lesions observed in patients with RHD (26). Later studies by Gorton et al. (30) reported valvulitis and myocarditis with infiltration of CD4+ cells, CD68+ macrophages and Anitschkow cells in the myocardium and mitral, aortic and tricuspid valves of Lewis rats following injection with recombinant M5 proteins (Table 1). Antibody and T cell responses to recombinant GAS M protein and the subsequent interactions with cardiac tissue have been predominantly investigated using a RAV model (Figure 1B) (30–34, 48). Furthermore, studies on Lewis rats indicated the role of infiltrating CD4+ cells and macrophages in the disease process. In addition to these histological changes, Lewis rats also demonstrated electrocardiographic and echocardiographic changes following exposure to killed GAS and recombinant GAS M proteins induce cardiac functional abnormalities comparable to patients with ARF (Figure 1D) (35, 36).

The hallmark features of ARF/RHD includes lesions in myocardium and valves (Figure 1E). In Lewis rats repeat injection with whole-killed GAS or recombinant GAS M proteins induced tissue cross-reactive antibodies and T cells (26, 27, 30, 31, 33, 34, 46, 48). Moreover, the involvement of Th-17 cells and associated regulators observed in the pathological process may potentially be considered as biomarkers for RHD (Figure 1B) (49, 50). In a separate experiment, in response to different streptococcal antigens, including both GAS and *Streptococcus dysgalactiae* subsp. *equisimilis* (SDSE/GGS), Lewis rats developed typical histological lesions with infiltration of inflammatory cells into cardiac tissue providing experimental evidence that streptococci other than GAS could trigger and/or exacerbate post-streptococcal carditis (Table 1). Lewis rats were also used to assess the preclinical immunogenicity and safety of a GAS M protein-based vaccine candidate (51, 52). Therefore, the Lewis rat model is not only useful in elucidating the pathophysiological mechanisms in ARF/RHD, but also provides an opportunity to identify, validate streptococcal epitopes that are truly pathogenic to ARF/RHD. It also enables the assessment of safety and efficacy of GAS antigen based prototype vaccine candidates (51, 52).

## Animal Models of Neurobehavioral Complications Associated With Streptococcal Infection

The two major neurobehavioral complications associated with post GAS infections are Sydenham chorea (SC) and pediatric autoimmune neuropsychiatric disorders associated with streptococcus (PANDAS) (53). SC is a neurological movement disorder described in ARF and is one of the major criterions for the diagnosis ARF (18). PANDAS is a sudden onset of obsessive-compulsive disorder (OCD) associated with GAS infection and not known to be clinically associated with ARF (54). The complex immunopathological mechanisms that mediated immune damage following GAS infections that leads to SC and PANDAS remain unclear (55). However, it has been shown that antibodies against GAS cross-react with neurotransmitter receptors (D1 and D2 dopamine receptors), signaling kinases and ion channels, located primarily in the basal ganglia of the brain in susceptible hosts due to molecular mimicry (56).

In the past many studies have been carried out to develop an animal model to investigate the post streptococcal neurobehavioral disorders (Table 1) (57–62). Initial experiments were carried out by infusion of serum from patients with suspected streptococcal related neuropsychiatric disorders directly in to the striatum of rats. However, not all such studies succeeded in modeling these stereotypic behaviors in mice and rats (63–66). In 2004, Hoffman et al. (57) injected female SJL/J mice with purified GAS M6 protein along with Freund's adjuvant and observed that a group of mice developed motor and behavioral problem. These investigators conducted another study by the passive transfer of sera from mice injected with GAS to naïve mice, which also developed in similar neurological and behavioral changes (58). In both these studies immunological analysis of the brain tissue showed anti streptococcal antibody deposition in deep cerebella nuclei and hippocampus.

A recent study by Brimberg et al. (59) observed neurobehavioral and immunological changes akin to SC and PANDAS in male Lewis rats following exposure to GAS antigens. Behavioral changes included impairment in handling food, traversing the narrow beam and obsessive-compulsive behavior (Figure 1C) (59). Lewis rats developed behavioral and neurological conditions similar to SC and PANDAS after passive transfer of serum from rats exposed to GAS infection (60). These studies showed elevated levels of antibodies against GAS M protein and cross-reactive antibodies against brain in the peripheral blood and brain, similar to antibodies present in SC and PANDAS patients (59, 60). Antibodies derived from GAS exposed animals have shown strong reactivity with D1 and D2 dopamine receptors and activated calcium/calmodulin-dependent protein kinase II signaling in brain tissue (59, 60). Similarly, *in vitro* studies demonstrated that monoclonal antibodies against N-acetyl-β-D-glucosamine and lysoganglioside GM1 induced the activity of calcium/calmodulin-dependent protein kinase II, which is potentially implicated as an important mediator of learning and behavior (56). Recent studies in C57BL/6, C57BL/6J, or SJL/J female mice following intranasal GAS challenge have demonstrated a breakdown in the Blood Brain Barrier (BBB) enabling the migration of GAS specific Th17 cells from nasal-associated lymphoid tissue to the brain, with the microglial activation and IgG deposition in the striatum (62, 67). Elevated levels of pro inflammatory cytokines including IL17A+ IFN-γ+ due to GAS autoimmunity disrupts the BBB to allow circulating autoantibodies and Th17 and Th1 cells to enter the brain, which targets neurons and trigger neurobehavioral changes (Table 1) (67, 68). In addition genetically modified mice lacking Th17 lymphocytes (SJL/J, RORγ*t*^+/GFP^ and RORγ*t*^GFP/GFP^ mice) have shown reduced BBB leakage, microglial activation, and antibody infiltration into the brain following intranasal challenge with GAS (Figure 1B). This demonstrates the importance of Th17 lymphocytes in BBB leakage and infiltration of autoantibodies into the brain tissue (67). Thus, rodent models are very useful for assessing the disease mechanisms associated with central nervous system to precisely determine sequential events following infection with GAS.

## Need for an Animal Model to Investigate Multiple Complications Associated With ARF/RHD

Post streptococcal autoimmune sequelae is a multisystem disorder affecting multiple organs including heart, brain, joints, connective tissues and skin (5). The immunopathology due to autoimmune response defers between organs. In the heart it is due to the pathological process initiated by the cross-reactive anti-GAS antibodies and T cells against host proteins (69). In the brain the disease is associated with IgG deposition (Figure 1F) (70). Whereas, in joints the pathogenesis is due to the immune complexes that bind to the synovial membrane and/or collagen in joints (5), and erythema marginatum might be due to cross-reactivity of anti-GAS antibody with keratin (71) and subcutaneous nodules might be due to a delayed hypersensitivity against GAS antigens (5). ARF patients can develop a combination of clinical symptoms that can lead to serious consequences. Approximately 30% of the patients with ARF can suffer from both cardiac and neurobehavioral complications (3). Moreover, due to the heterogeneity of ARF/RHD, an animal model might reflect a specific phenotype of the diverse complications from those observed in human disease. Therefore, an animal model which can reflect both cardiac and neurobehavioral conditions would be a remarkable advancement in ARF/RHD research, not only to investigate the pathophysiology but also to assess the safety and efficacy of vaccine candidates and treatment modalities. Furthermore, in compliance with more stringent animal welfare considerations (e.g., 3Rs rules', for **r**eplacement, **r**eduction and **r**efinement) determining different aspects of a disease in a single animal will minimize the number of animals needed for research (Figure 1). Recently we have achieved this goal by modeling both cardiac and neurobehavioral changes in Lewis rats and rats injected with GAS shown impairments in fine motor control, gait and balance and obsessive-compulsive behavior similar to SC and PANDAS together with functional and immunological changes previously observed in the RAV model (72). Moreover, post-streptococcal complications including RHD and neurobehavioral changes such as SC are prominent in females (10, 73–75), thus most of the studies on RHD have been conducted in female mice or rats. However, neurobehavioral studies described in the literature have either been conducted on male or female mice but solely on male rats. Our recent observations demonstrated that there were no significant difference in using both genders of Lewis rats to simultaneously model carditis and neurobehavioral changes (72). To further validate multiple complications associated with ARF/RHD, more studies are warranted on the Lewis rat model.

## Limitations of ARF/RHD Animal Models

While significant advances in animal models of ARF/RHD have been made in the last decade, there is still a paucity in pre-clinical studies on other complications associated with ARF/RHD including neurobehavioral changes, arthritis and skin manifestations. Arthritis is observed in ~50–70% of patients with ARF and is a major Jones Criterion for the diagnosis of ARF. However, none of the animal studies have investigated GAS induced autoimmune process in subcutaneous tissue and joint tissue in any of these models.

## Conclusion

Laboratory models are important to determine the early events leading to chronic disease. In particular when clinical studies are not possible during the early stages. In a credible animal model symptoms of physiological, anatomical and behavioral conditions must be comparable to those observed in human disease. In addition, an animal model should be reliable and the changes observed must be reproducible across laboratories. An animal model of ARF/RHD and associated neurobehavioral complications should possess functional and pathological changes encompassing motor deficits as well as compulsive and stereotyped behaviors similar to SC. Since genotypes, sex and age difference affects the development of autoimmune complication; selection of an appropriate animal model is important to investigate the pathogenesis of ARF/RHD and associated complications. Several animal models have been tested to investigate the onset, and progression of ARF/RHD. The Lewis rat model characterized by us and others, is a reliable model to investigate early events that lead to cardiac valvular pathology. Together with advances in novel imaging technologies and integrated computational approaches our model will provide the means to address these challenges (76). Importantly, comparison of experimental results with clinical observations to extrapolate the sequential event that follow infection with GAS leading to autoimmune complications requires prudence and caution.

## Author Contributions

RAMR, SS, ASH, NMA, KSS, DJM, and NK wrote the main manuscript. All authors have read and approved the manuscript.

## Conflict of Interest

The authors declare that the research was conducted in the absence of any commercial or financial relationships that could be construed as a potential conflict of interest.
